# Migration Properties Distinguish Tumor Cells of Classical Hodgkin Lymphoma from Anaplastic Large Cell Lymphoma Cells

**DOI:** 10.3390/cancers11101484

**Published:** 2019-10-02

**Authors:** Olga Goncharova, Nadine Flinner, Julia Bein, Claudia Döring, Emmanuel Donnadieu, Sandy Rikirsch, Marco Herling, Ralf Küppers, Martin-Leo Hansmann, Sylvia Hartmann

**Affiliations:** 1 Dr. Senckenberg Institute of Pathology, Goethe University Frankfurt, 60590 Frankfurt am Main, Germany; 2 Institute of Informatics/Frankfurt Institute for Advanced Studies, Goethe University, 60438 Frankfurt am Main, Germany; 3 Inserm, U1016, Institut Cochin, CNRS, UMR8104 and Université Paris Descartes, F-75014 Paris, France; 4 The Laboratory of Lymphocyte Signaling and Oncoproteome, Department I of Internal Medicine, Center for Integrated Oncology (CIO) Aachen-Bonn-Cologne-Duesseldorf, CECAD and CMMC, University of Cologne, 50937 Cologne, Germany; 5 Institute of Cell Biology (Cancer Research), University of Duisburg-Essen, 45122 Essen, Germany; 6 Reference and Consultant Center for Lymph Node and Lymphoma diagnostics, 60590 Frankfurt, Germany; 7 Frankfurt Institute of Advanced Studies, 60438 Frankfurt am Main, Germany

**Keywords:** anaplastic large cell lymphoma, dissemination, rosetting T cells, classical Hodgkin lymphoma, chemokine receptors, cell motility, segmentation, image analysis, gene expression

## Abstract

Anaplastic large cell lymphoma (ALCL) and classical Hodgkin lymphoma (cHL) are lymphomas that contain CD30-expressing tumor cells and have numerous pathological similarities. Whereas ALCL is usually diagnosed at an advanced stage, cHL more frequently presents with localized disease. The aim of the present study was to elucidate the mechanisms underlying the different clinical presentation of ALCL and cHL. Chemokine and chemokine receptor expression were similar in primary ALCL and cHL cases apart from the known overexpression of the chemokines CCL17 and CCL22 in the Hodgkin and Reed-Sternberg (HRS) cells of cHL. Consistent with the overexpression of these chemokines, primary cHL cases encountered a significantly denser T cell microenvironment than ALCL. Additionally to differences in the interaction with their microenvironment, cHL cell lines presented a lower and less efficient intrinsic cell motility than ALCL cell lines, as assessed by time-lapse microscopy in a collagen gel and transwell migration assays. We thus propose that the combination of impaired basal cell motility and differences in the interaction with the microenvironment hamper the dissemination of HRS cells in cHL when compared with the tumor cells of ALCL.

## 1. Introduction

Anaplastic large cell lymphoma (ALCL) is a CD30-expressing malignant lymphoma of T-cell origin [1,2]. ALCL is divided into ALK-positive and ALK-negative subtypes, based on the presence of the chromosomal nucleophosmin-anaplastic lymphoma kinase (NPM-ALK) or variant translocation [3,4]. The cytomorphological hallmark of ALCL is the presence of tumor cells with large kidney shaped nuclei and abundant cytoplasm [5,6]. Although sequencing the T-cell receptor revealed the clonal T-cell origin of ALCL [7], the expression of most T-cell markers is lost in this lymphoma [8].

Classical Hodgkin lymphoma (cHL) is another CD30-expressing malignant lymphoma [9] that shares morphological and phenotypical features with ALCL and can impose a difficult differential diagnosis. In contrast with ALCL, the Hodgkin and Reed-Sternberg (HRS) cells of cHL are derived from pre-apoptotic germinal center B cells [10,11] The expression of most B-cell markers is generally lost in cHL [12].

Despite the number of similarities between ALCL and cHL, there are important differences in their clinical behaviors. cHL frequently occurs as a localized disease that can often be successfully treated [13]. In contrast, ALCL is characterized by the relatively early and aggressive spread to distant sites and is frequently diagnosed at an advanced stage, then with a rather unfavorable prognosis [14].

Several factors can influence the dissemination of lymphoma cells, including chemokines and cytokines, which can trigger the process of chemotaxis. Several studies have been performed to investigate the expression of chemokine receptors and their ligands in the HRS cells of cHL [15,16,17,18,19,20] These studies found that the chemokine receptors CCR5, CXCR3, CXCR4, CXCR5 and CCR7 are expressed in the HRS cells of cHL in a significant number of cases [15,18,19]. Furthermore, the ligands CXCL10, CXCL12, CXCL13, CCL21, CX3CL1, CCL5 and CCL17 are expressed in cHL cell lines and primary HRS cells [17,18,19,21,22]. CCL17 and CCL5 are capable of attracting T helper 2 (Th2) and regulatory T (Treg) cells, which interact with HRS cells and form what has been named rosettes around them [17,23]. Compared with cHL, only a few studies on chemokines and their receptors have been conducted in ALCL cells. The chemokine receptors CCR1, CXCR3, CCR4 and CCR5 were found to be heterogeneously expressed in ALCL tumor cells [24,25,26,27].

Interactions with cells of the microenvironment can influence the migration behaviors of lymphoma cells. To date, little is known regarding the tumor microenvironment in ALCL. In contrast, the microenvironment in cHL has been extensively studied. HRS cells constitute approximately 1% of the cellular infiltrate in cHL tumor tissue and can actively shape their microenvironments, which is primarily composed of T cells, macrophages, eosinophils and neutrophils [28,29].

Intrinsic migration properties can also influence lymphoma cell dissemination in the human body. Normal T cells are slightly faster than normal B cells, as peak velocities for T cells up to 30 µm/min and for B cells up to 20 µm/min have been observed [30]. For the HRS cells of cHL, migration depends on active Wnt signaling [31]. Wnt signaling is also active in a subpopulation of Karpas-299 and SupM2 ALCL cell lines [32]. Thus, Wnt signaling is likely important for the migration of both lymphoma types.

The aim of the present study was thus to clarify the primary factors that contribute to the differential dissemination behaviors between ALCL and cHL.

## 2. Results

### 2.1. Expression of Chemokine Receptors is Heterogeneous in both ALCL and cHL

**Figure 1:**
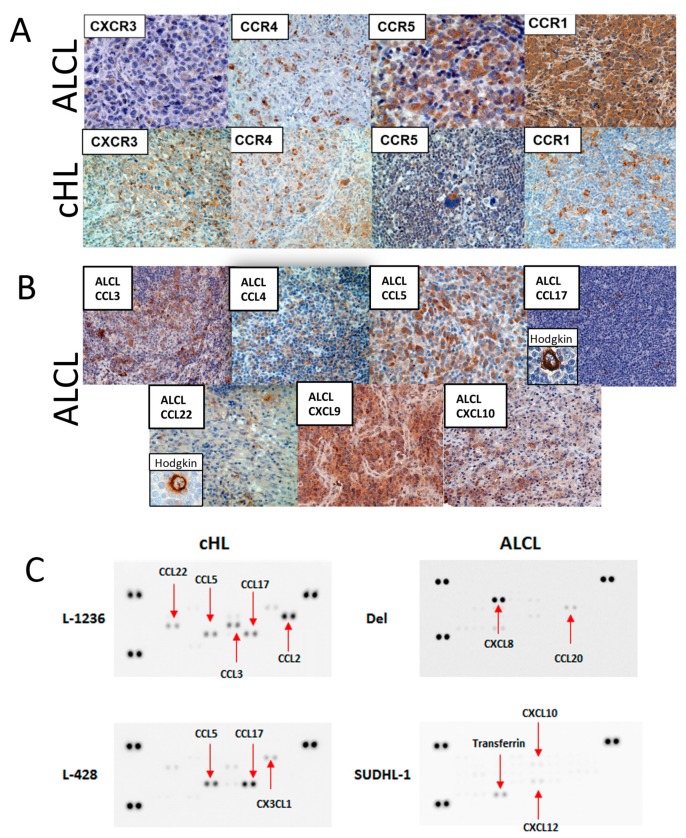
Chemokine receptor and ligand expression in primary cases of ALCL and cHL mixed cellularity as well as cell lines L-1236, L-428, DEL and SU-DHL-1. (**A**). Representative examples of chemokine receptor expression of CXCR3, CCR4, CCR5 and CCR1 in cases of ALCL and cHL. (**B**). Apart from CCL17 and CCL22, which are negative in ALCL, most corresponding chemokines are expressed in cases of ALCL. Inserts show the typical strong expression of CCL17 and CCL22 in cHL. (**C**). Examples of chemokine arrays showing strong secretion of CCL17 in cHL cell lines L-1236 and L-428 in contrast to ALCL cell lines DEL and SU-DHL-1.

To investigate whether the dissemination of lymphoma cells is dependent on the expression of chemokine receptors, we examined the expression of the chemokine receptors CXCR3, CCR4, CCR5 and CCR1 in primary cases of ALCL and cHL by immunohistochemistry. Chemokine receptors were chosen based on the hypothesis that ALCL tumor cells would be more similar to reactive T cells in chemokine receptor expression than HRS cells of cHL. These receptors are typically expressed by Th1, Th2 and memory T cells [33]. CXCR3 was expressed in the tumor cells in 8/13 ALK^−^ ALCL cases (61%), 6/11 ALK^+^ ALCL cases (55%) and 5/11 cHL cases (45%) (Figure 1A, Supplementary Table S1). CCR4 was positive in the tumor cells of 9/15 ALK^−^ ALCL cases (60%), 10/15 ALK^+^ ALCL cases (67%) and 6/10 cHL cases (60%). CCR5 was expressed in 17/22 ALK^−^ ALCL cases (77%), 12/16 ALK^+^ ALCL cases (75%) and 6/8 cHL cases (75%). CCR1 was expressed in 1/23 ALK^−^ ALCL cases (4%), 3/13 ALK^+^ ALCL cases (23%) and 4/10 of cHL cases (40%). Thus, the expression of these selected chemokine receptors appears to be comparable between ALCL and cHL tumors, and they are not likely to contribute to the different dissemination patterns clinically observed in both lymphomas.

Additionally, the expression of the chemokine receptors CXCR3, CCR4, CCR5 and CCR1 was characterized in five ALCL and four cHL cell lines (Supplementary Table S2). Among ALCL cell lines, CCR5 was clearly expressed in the SU-DHL-1 and MAC-1 cell line, and CXCR3 was expressed in the SR-786 cell line. The cHL cell lines L-428 (nodular sclerosis cHL), L-1236 (mixed cellularity cHL) and KM-H2 (mixed cellularity cHL) and the ALCL cell line MAC-1 were positive for CCR4 (Supplementary Table S2). Thus, the expression of chemokine receptors appears to be downregulated in lymphoma cell lines when compared with their expression in primary lymphoma samples.

### 2.2. CCL17 and CCL22 are Frequently Expressed by cHL But Only Very Rarely in ALCL Tumor Cells

Using immunohistochemistry in primary ALCL and cHL cases, we evaluated the expression of the chemokines that bind to the receptors that we analyzed above (Figure 1B; Supplementary Table S3). The CXCR3 ligands CXCL9 and CXCL10 were more frequently expressed in ALCL cases (75% and 44%, respectively) than in cHL cases (40% and 25%, respectively) (Supplementary Table S3) [17,34]. CCL5 (ligand for CCR5 and CCR1) was more frequently expressed in the HRS cells of cHL cases (86%) than in ALCL cases (24%) (Supplementary Table S3). A striking difference between ALCL and cHL cases was observed for CCL17 (0% of ALCL versus 85% of cHL cases) and CCL22 expression (0% of ALCL versus 80% of cHL) (Supplementary Table S3), which confirms previously reported data [17]. In line with the observations for primary cases, the cHL cell lines L-428 and L-1236 strongly expressed CCL17, and L-1236 also secreted CCL22 (Supplementary Table S4; Figure 1C). In contrast, none of the ALCL cell lines investigated (SU-DHL-1, Del, MAC-1, SR-786, KARPAS-299, and TS-G1; Supplementary Table S4) secreted either CCL17 or CCL22. Therefore, consistent with previous studies [17], CCL17 and CCL22 expression appears to be exclusive to cHL primary tumor cells and cell lines and likely represents a specific feature of this lymphoma over ALCL. Consistent differences in CCR expression between ALK^+^ and ALK^−^ ALCL were not observed.

### 2.3. cHL Has a More Abundant Microenvironment and HRS Cells Are More Effective for the Induction of T-Cell Rosettes Compared with ALCL

**Figure 2:**
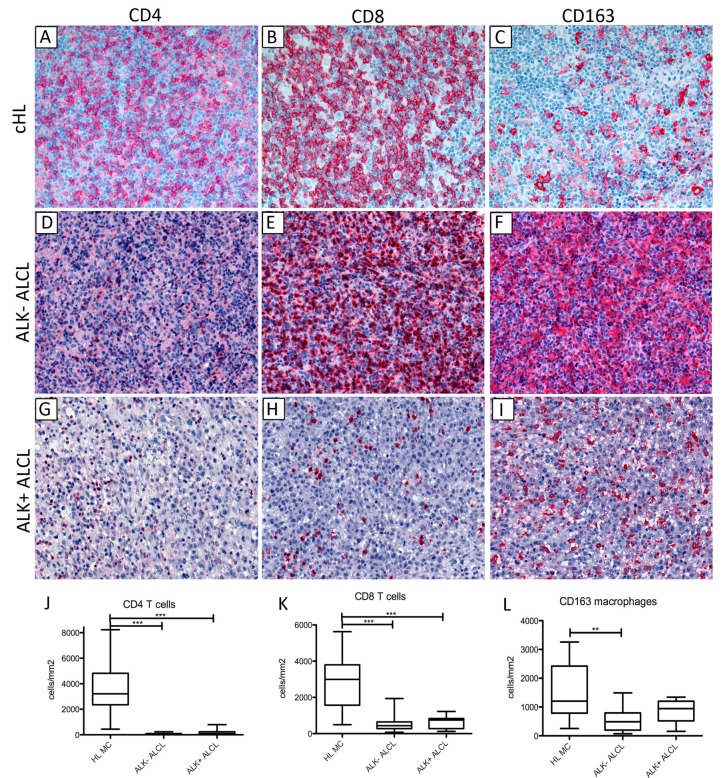
Composition of the microenvironment differs between ALCL and cHL. (**A**,**D**,**G**) Examples of CD4-immunostainings (200× magnification) in cHL (mixed cellularity subtype), ALK^−^ and ALK^+^ ALCL with higher numbers of CD4-positive T cells in the cHL case. (**B**,**E**,**H**) Examples of CD8-immunostainings (200× magnification) in cHL (mixed cellularity subtype), ALK^−^ and ALK^+^ ALCL with the highest number of CD8-positive T cells in the cHL case. (**C**,**F**,**I**) Examples of CD163-immunostainings (200× magnification) in cHL (mixed cellularity subtype), ALK^−^ and ALK^+^ ALCL. In this example the ALK^−^ ALCL case has the highest number of CD163-positive macrophages. (**J**). Quantification of CD4-positive T cells/mm² revealing significantly higher numbers of CD4-positive T cells in the microenvironment of mixed cellularity cHL cases (n = 15) when compared with ALK^+^ (n = 10) and ALK^−^ ALCL (n = 12) (Mann-Whitney test, *** *p* < 0.001). Medians, minimums and maximums are plotted. (**K**). Quantification of CD8-positive T cells/mm² revealing significantly higher numbers of CD8-positive T cells in the microenvironment of mixed cellularity cHL cases (n = 15) when compared with ALK^+^ (n = 11) and ALK^−^ ALCL (n = 14) (Mann-Whitney test, *** *p* < 0.001). Medians, minimums and maximums are plotted. (**L**). Quantification of CD163-positive macrophages/mm². CD163-positive macrophages were significantly more abundant in the microenvironment of mixed cellularity cHL cases (n = 15) when compared with ALK^−^ ALCL (n = 13) (Mann-Whitney test, ** *p* < 0.01). The difference observed in ALK^+^ ALCL (n = 9) was not significant. Medians, minimums and maximums are plotted.

**Figure 3:**
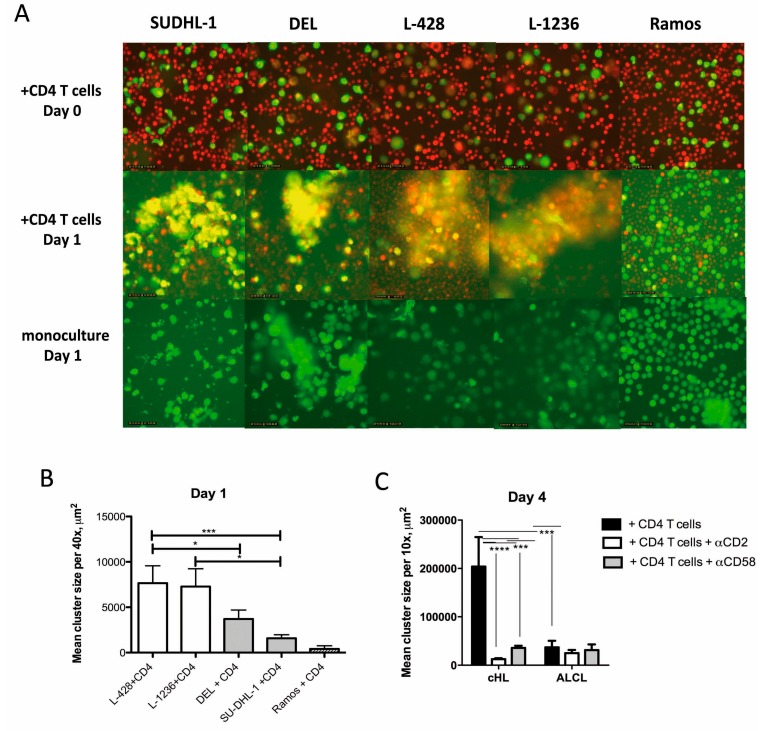
Clusters with CD4-positive T cells are more prominent with cHL cell lines L-1236 and L-428 when compared with ALCL cell lines DEL and SU-DHL-1. (**A**). Examples of clusters in the different cell lines on day 1 compared with monoculture of the corresponding cell lines. When CSFE-labeled tumor cells get in contact with red-labeled CD4^+^ T cells, a yellow color is created by overlay. The Burkitt lymphoma cell line Ramos was used as a negative control. (**B**). Mean cluster size on day 1. Clusters observed with the cHL cell lines L-428 and L-1236 were larger when compared with ALCL cell lines DEL and SU-DHL-1 (Mann-Whitney-test, * *p* < 0.05, *** *p* < 0.001). Means and standard error of means of four independent experiments with three to five images per experiment, 40× magnification. (**C**). Mean cluster size at day 4 with and without addition of blocking antibodies against CD2 and CD58. Clusters with the cHL cell lines L-1236 and L-428 are significantly larger than those seen with ALCL cell lines DEL and SU-DHL-1 at baseline conditions. Addition of either blocking antibody anti-CD2 or anti-CD58 resulted in a significantly reduced cluster size in cHL cell lines (one-way Anova with Bonferroni´s multiple comparison test. *** *p* < 0.001). Means and standard error of means of three images per experiment in three independent experiments, 10× magnification.

Because we observed differences in CCL17 and CCL22 expression between ALCL and cHL cells and because both of these chemokines are important factors for the attraction of T cells to the microenvironment, we systematically assessed the microenvironmental composition of ALCL and cHL cases, as the tumor microenvironment can impair or promote the active movement and dissemination of lymphoma cells [35]. Although cHL is known for an abundant reactive microenvironment, a detailed characterization of the microenvironment of ALCL has not been performed. Thus, the numbers of CD4^+^ and CD8^+^ T cells and CD163^+^ macrophages were quantified in ALK^+^ or ALK^−^ ALCL cases and compared with the numbers in cHL cases. The mixed cellularity subtype of cHL was chosen as comparison since it is most similar to ALCL and can sometimes be a difficult differential diagnosis. Both CD4 and CD8 T cells were present at lower frequencies in ALCL cases when compared with cHL cases (Figure 2). The amount of CD163^+^ positive macrophages was only slightly reduced in ALCL cases compared with cHL cases, with some ALCL and cHL cases showing comparable amounts of CD163^+^ macrophages (Figure 2). The phenomenon of CD4^+^ T cells rosetting around HRS cells is well known in cHL, whereas T-cell rosettes have not been observed in ALCL. Using a co-culture system where ALCL (DEL, SU-DHL-1), cHL (L-428, L-1236) or Burkitt lymphoma (Ramos) cell lines were cultured together with CD4^+^ T cells isolated from peripheral blood, we studied rosette formation capacity in vitro. On day 1 after the addition of T cells, we observed that cHL cell lines formed clusters with CD4^+^ T cells more efficiently than ALCL cell lines (Figure 3A,B), whereas almost no clusters were formed in the negative control co-cultured with the Burkitt lymphoma cell line Ramos. When we extended the observation until day 4 and applied anti-CD2 and anti-CD58 antibodies, which can block cell-cell interactions via adhesion molecules, cluster formation was reduced in cHL cell lines co-cultured with CD4^+^ T cells (Figure 3C). In conclusion, these data confirm that the microenvironment for cHL includes more T cells and macrophages than that for ALCL.

Our results also implicate that HRS cells are more effective for the induction of T-cell rosettes than ALCL cells, and that adhesion molecules contribute to the interaction between HRS cells and T cells.

### 2.4. Filopodia-like Structures Occur More Frequently and Are Longer in the HRS Cells of cHL Cell Lines Than Those in ALCL Cell Lines

Filopodia are thin, finger-like and highly dynamic actin-rich membrane protrusions that extend out from the cell edge [36]. They are required for the cellular movement and for sensing the environment. Actin filaments are arranged in a parallel manner in filopodia extending in an orthogonal way from the cell center [37]. During cell migration, actin filaments can also polymerize in a more flat, sheet-like manner, called lamellipodia [37]. Because we observed important differences both in the composition of the microenvironment and in the interactions among ALCL and cHL tumor cells with their respective microenvironments, we assessed whether there are structural differences in the basal cell shape or the cytoskeleton composition between ALCL and cHL cell lines in monoculture by transducing the cells with Life-Act-GFP to fluorescently label actin filaments. Static, 3D, Z-stack images were acquired from the cell lines DEL, SU-DHL-1, L-1236 and L-428, embedded in a collagen gel using a spinning disc microscope, and the image segmentation.

**Figure 4:**
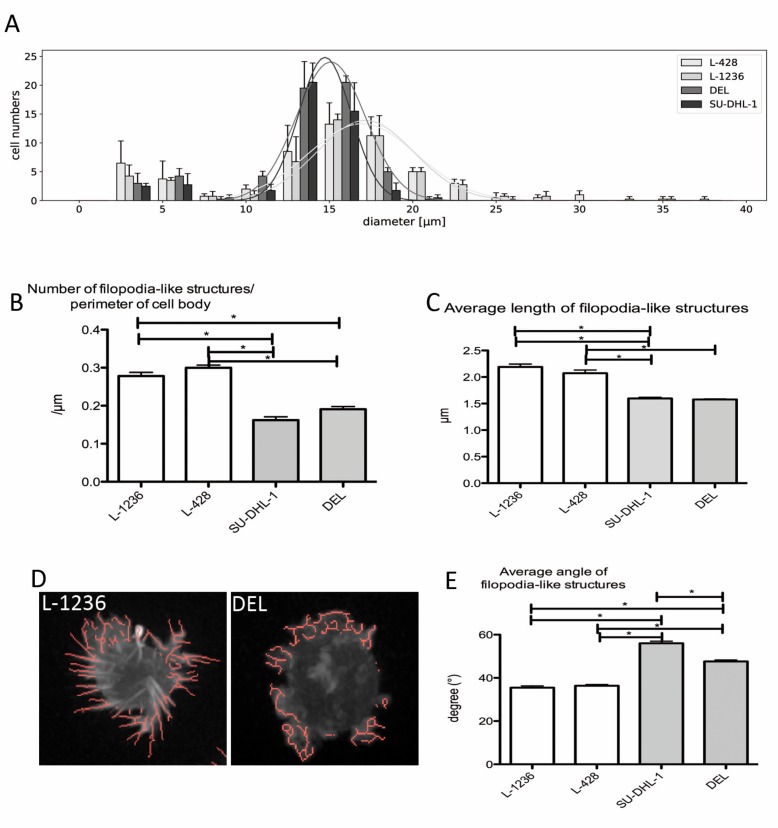
Filopodia-like structures are more frequent, longer and have a more orthogonal orientation in cHL cell lines L-1236 and L-428 compared with ALCL cell lines DEL and SU-DHL-1, which are embedded in a collagen gel. (**A**). Size distribution of the cell lines is wider in L-1236 and L-428 with on average larger cells compared with ALCL cell lines SU-DHL-1 and DEL. Cell sizes were determined in in 36–56 GFP-Life-Act-labeled cells in each of four independent experiments by spinning disk microscopy. Means and standard deviation. (**B**). The number of filopodia-like structures/perimeter of the main cell body is significantly higher in the cHL cell lines L-1236 and L-428 when compared with the ALCL cell lines SU-DHL-1 and DEL (Mann-Whitney-U-test, * *p* < 0.05). Small fragments < 10 µm were devoid of nuclear material, thus probably representing large oncosomes atypically large extracellular vesicles observed in cancer cell lines. A total of 36–56 GFP-Life-Act-labeled cells were analyzed each in four independent experiments by spinning disk microscopy. Means and standard error of means are plotted. (**C**). The average length of filopodia-like structures is significantly higher in both cHL cell lines L-1236 and L-428 when compared with ALCL cell lines SU-DHL-1 and DEL (Mann-Whitney-U-test, * *p* < 0.05). A total of 36–56 GFP-Life-Act-labeled cells were analyzed each in four independent experiments by spinning disk microscopy. Means and standard error of means are plotted. (**D**). Examples of the segmentation analysis of filopodia-like structures in the cell lines L-1236 and DEL. (**E**). The mean angle between filopodia-like structures and the orthogonal axis to the cell center is significantly lower in cHL cell lines L-1236 and L-428 when compared with with ALCL cell lines SU-DHL-1 and DEL, indicating a more straight orientation of filopodia-like structures in the cHL cells (Mann-Whitney-U-test, * *p* < 0.05). A total of 36–56 GFP-Life-Act-labeled cells were analyzed each in four independent experiments by spinning disk microscopy. Means and standard error of means are plotted.

The mean size of tumor cells was slightly larger in the cHL cell lines L-1236 and L-428 compared with the ALCL cell lines DEL and SU-DHL-1. The size distribution of all four cell lines was fitted to a Gaussian distribution, and cHL cell lines were found to have a broader distribution than ALCL cell lines (Figure 4A), after excluding small cell fragments of < 10 µm (Figure 4A), probably presenting cancer-specific atypically large extracellular vesicles, also called oncosomes [38].

Because we aimed to analyze resting cells using this approach, the cell polarity was determined, and there were no general differences among the four cell lines, with the majority of cells representing unpolarized cells. Filopodia-like structures were recognized as Life-Act-GFP positive structures emerging from the cell membrane. The numbers of Life-Act-positive filopodia-like structures emerging from the cell body were significantly higher in the cHL cell lines L-1236 and L-428 than in the ALCL cell lines DEL and SU-DHL-1 (*p* < 0.05, Mann-Whitney U-test, Figure 4B). Furthermore, the lengths of the filopodia-like structures were significantly longer in the cHL cell lines L-1236 and L-428 than in the ALCL cell lines DEL and SU-DHL-1 (Figure 4C and Supplementary Figure S1, Supplementary Movies S1 and S2). The filopodia-like structures in the cHL cell lines were more frequently orientated in an orthogonal manner than in the ALCL cell lines (median angles 38.7° and 37.2° in L-428 and L-1236, respectively, compared with 45.8° and 52.0° in DEL and SU-DHL-1, respectively; Figure 4D,E). Thus, the configuration of the filopodia-like structures in cHL cell lines strongly resembles typical filopodia reflecting microenvironment sensing. In contrast, the filopodia-like structures in ALCL cell lines showing crossing actin fibres closely resemble lamellipodia and are thus likely more effective for cell migration.

### 2.5. ALCL Cells Actively Migrate Towards an FCS Gradient and Move Faster in Bovine Collagen Type I Gel than HRS Cells

Because we observed differences in the numbers, lengths and orientations of filopodia between cHL and ALCL cell lines, we assessed whether these cell lines also differ in their intrinsic abilities to move.

To examine whether cHL and ALCL cells employ different migration approaches that might be responsible for differences in basic cell motility, a collagen degradation assay was performed. For this purpose, cell lines were embedded in a collagen gel and allowed to migrate over night. Degraded collagen was consequently detected by an antibody against cleaved collagen (Supplementary Methods). The fibrosarcoma cell line HT-1080 was used as a positive control and demonstrated the strong degradation of the collagen matrix. In contrast, the lymphoma cell lines L-1236, L-428, DEL and SU-DHL-1 degraded collagen to a much lower degree than HT-1080 (Supplementary Figure S2), with significant collagen degradation observed for the ALCL cell lines DEL and SU-DHL-1 (Mann-Whitney U-test compared with negative control, *p* < 0.05). Thus, the ALCL and cHL cell lines both primarily present an amoeboid-like movement and make only little use of collagen degradation. In HRS cell lines, several factors that contribute to the degradation of the extracellular matrix are downregulated [39].

**Figure 5:**
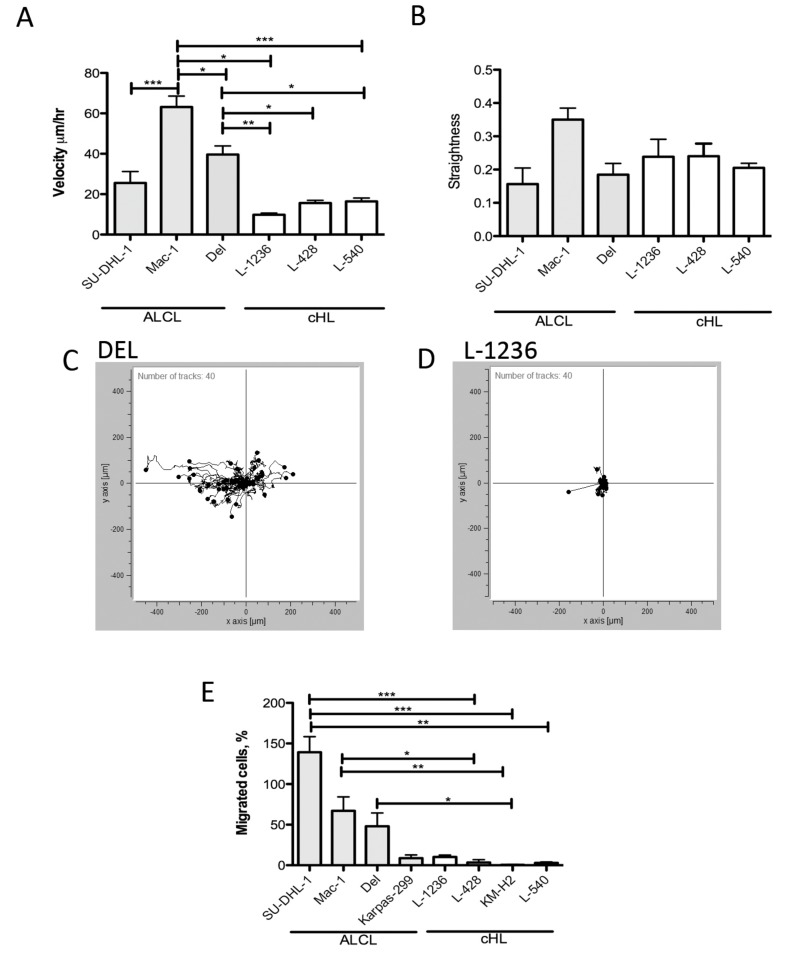
Intrinsic migration properties of ALCL and cHL cell lines in collagen gel and transwell chambers with FCS gradient. (**A**). Basic velocity determined by tracking of time lapse microscopy of ALCL and cHL cell lines embedded in a collagen type I gel in Ibidi chemotaxis slides (* *p* < 0.05, ** *p* < 0.01, *** *p* < 0.001, one-way-Anova, with a Bonferroni-Holm p-value adjustment for multiple comparisons). Means and standard error of means of three independent experiments. (**B**). Straightness (euclidean distance/accumulated distance) of cell movements in a collagen type I gel in Ibidi chemotaxis slides determined by tracking of time lapse microscopy. Means and standard error of means of three independent experiments. (**C**). Example of tracks of the ALCL cell line DEL in bovine collagen type I gel in Ibidi chemotaxis slides determined by tracking of time lapse microscopy. (**D**). Example of tracks of the cHL cell line L-1236 in bovine collagen type I gel in Ibidi chemotaxis slides determined by tracking of time lapse microscopy. (**E**). Efficiency of migration versus an FCS gradient of ALCL and cHL cell lines in an uncoated transwell of 8 µm pore size. ALCL cell lines showed a higher migration efficiency, which was significantly higher for SU-DHL-1, MAC1 and DEL compared with L-1236 (* *p* < 0.05, ** *p* < 0.001, *** *p* < 0.0001, Kruskal-Wallis-test). Means and standard error of means of three independent experiments.

Furthermore, intrinsic cell motility properties were studied in a bovine collagen type I gel. Basal random cell motility was monitored in a gel using time-lapse microscopy over a 24-hour period. The ALCL cell lines SU-DHL-1, DEL and MAC-1 generally showed higher velocities than the cHL cell lines L-1236, L-428 and L-540 (Figure 5A,C,D), with MAC-1 cells being the fastest. Furthermore, the straightness (Euclidean distance/accumulated distance) of all cell lines was evaluated. No differences in straightness were observed among the ALCL and cHL cell lines (Figure 5B).

In an independent approach, cHL and ALCL cell lines were assessed for their migration potential by using an 8 µm pore-size transwell chamber with an FCS gradient. Similar to the experiments in bovine collagen type I, the ALCL cell lines migrated though the transwell membrane more efficiently than the cHL cell lines (Figure 5E).

## 3. Discussion

The aim of the present study was to determine the mechanisms that underly the different clinical presentations of cHL, a frequently limited disease, and ALCL, which presents with a more advanced spread, despite the fact that these two lymphomas can present with overlapping histopathological and morphological characteristics.

In line with previous studies, we confirmed that the tumor cells from primary cases of both ALCL and cHL commonly express several chemokine receptors, with high variability between individual cases [25,27]. We did not observe consistent differences in chemokine receptor expression among ALK^+^ ALCL cases, ALK^−^ ALCL cases, and cHL cases. Notably, the expression of chemokine receptors was downregulated in the majority of ALCL and cHL cell lines, in line with prior gene expression profiling data [39]. This finding is likely because chemokine signaling is associated with microenvironmental cell interactions, which are lacking in cell cultures. However, immunohistochemical expression analysis of chemokine receptors in primary cases and cell lines was unable to explain the differences observed in the spreading patterns of these two lymphomas.

Studiing the expression of chemokines, we additionally found a high degree of similarity between ALCL and cHL cells for most chemokines, including CCL3, CCL4, CCL5, CXCL9 and CXCL10. In contrast, the expression of CCL17 and CCL22 was highly specific to the HRS cells of cHL, as has been described previously [23,40,41]. In contrast with our results, in previous studies, ALK^−^ ALCL have been noted to express CCL17 in some cases (1/27 ALCL cases in Peh et al. [42] and 12/27 ALK^−^ ALCL cases in Vermeer et al. [43]). In the present study, we only found CCL17 expression in a small fraction of tumor cells in 3/35 ALK^−^ ALCL cases. Because the distinction between cHL and ALK^−^ ALCL cells was unreliable prior to the availability of PAX5 immunostaining, CCL17-expressing ALK^−^ ALCL cells may have been misclassified as cHL cells in some older studies. Thus, CCL17 is likely specifically expressed by the HRS cells of cHL. Likewise, CCL22 is specifically expressed in the HRS cells of cHL and not in ALCL cells [23,44,45]. Genomic loci coding for both chemokines can present copy number amplifications in cHL [46] and may thus lead to an overexpression of these chemokines in cHL. As previously described, HRS cells can shape their microenvironment through the expression of CCL17 and CCL22, attracting CCR4-expressing CD4-positive T cells, which then form rosettes around the HRS cells [17,40,47,48,49]. Therefore, the strong expression of these chemokines correlates well with the higher amounts of CD4-positive T cells and macrophages, which are both attracted by CCL17 and CCL22 and occur at higher frequency in the microenvironment of cHL cases compared with ALCL cases. When the ALCL cell line Karpas-299 was genetically modified to overexpress CCL17, it was able to attract CCR4-positive T cells at a level comparable to those of cHL cell lines [50]. Therefore, differences in the composition of the microenvironment between ALCL and cHL cases are likely associated with the secretion of CCL17 and CCL22 by the HRS cells thus confirming previously published data [23,40,41,42]. Similiarly, we could also confirm the more efficient induction of T-cell rosettes by cHL cell lines [51,52], which can be blocked by the addition of anti-CD2 or anti-CD58 antibodies, in line with previous observations that adhesion molecules play important roles in the formation of T-cell rosettes around HRS cells [52,53,54,55]. We assumed the effects of HLA-mismatch to be negligible at these early time points since an anti-HRS cell activity of CD4^+^ T cells against HL cell lines in coculture became detectable only after about 6 days of culture [56]. With regard to the dissemination of the lymphoma cells, tumor cells that adhere to T lymphocytes, such as HRS cells, should be less able to migrate than a pure tumor cell population, such as ALCL cells.

However, in addition to the influence of microenvironmental cells on the lymphoma dissemination, we also observed important differences in the intrinsic motility of cHL and ALCL cell lines, which have not been described to date. Generally, despite some heterogeneity between the cell lines, ALCL tumor cells migrated more efficiently in a collagen gel and towards an FCS gradient in transwell chambers. This is in line with the ineffective migration of cHL cell lines L-1236 and HDLM-2, which has been previously described in a transwell approach [57]. Differences in the actin skeleton between cHL and ALCL cell lines likely contribute to these differences in migration behavior, since actin structures in ALCL cell lines were oriented similar to lamellipodia, whereas actin structures in cHL cell lines resembled filopodia. Studies by Ambrogio et al. [58] and Colomba et al. [59] found a deregulation of *CDC42* and *RAC1* in ALK+ ALCL, which is in line with the enhanced motility of ALCL tumor cells, observed in the present study.

In summary, several features contribute to the impaired motility observed for HRS cells compared with ALCL cells. We here confirm the secretion of the chemokines CCL17 and CCL22 resulting in the attraction of T cells, which are closely attached to HRS cells by adhesion molecules. The larger sizes of HRS cells, combined with the surrounding T-cell rosette, can restrain the dissemination of these cells in the preformed lymphoid sinuses of a lymph node. Furthermore, we could for the first time demonstrate differences in intrinsic basal cell motility between cHL and ALCL cell lines contributing to a less efficient spread of HRS cells than ALCL cells. The molecular mechanisms underlying this feature should be further investigated in future studies.

## 4. Materials and Methods

Detailed information can be found in the Supplementary Methods section (Supplementary File S1).

### 4.1. Immunohistochemistry

Immunohistochemical stainings were performed as described previously [60]. The antibodies used, their providers, and the dilutions used are provided in the Supplementary Methods. Cases representing cHL mixed cellularity (MC) (n = 11), ALK^+^ ALCL (n = 16) and ALK^−^ ALCL (n = 23) were retrieved from the archives of the Dr. Senckenberg Institute of Pathology. Sample sizes were based on availability of tissue samples. Samples not matching the appropriate diagnostic criteria were excluded from the study. The ethics committee of the University Hospital Frankfurt approved this study (No. 287/17), and the informed consent of the patients was obtained in accordance with the declaration of Helsinki. All cases were reviewed by the authors S.H. and M.L.H., and diagnoses were confirmed according to the 2017 WHO classification [1] for all cases. Immunohistochemical stainings were scored as being positive when at least 50% of the tumor cells showed distinct staining.

### 4.2. Human Chemokine Array

ALCL and cHL cells were cultured under standard conditions. MAC-1 was obtained from one of the authors (M.H.). All other cell lines were purchased from the German Collection of Microorganisms and Cell Cultures (Braunschweig, Germany) and were regularly tested for mycoplasma contamination. All cell lines were authenticated by STR profiling. After 48 hours, supernatants were analyzed with a human chemokine array kit (R&D Systems, Abingdon, UK), according to manufacturer’s instructions. The membranes were exposed for 10 minutes using a Fusion SL Vilber Lourmat device (Peqlab, Erlangen, Germany). One array per cell line was analyzed.

### 4.3. Analysis of Cell Motility in A Collagen Type I Gel

After gelation of a 1.5 mg/mL bovine collagen type I gel (Advanced Biomatrix, San Diego, CA, USA), the left and right chambers of an Ibidi µ-slide chemotaxis (Ibidi, Martinsried, Germany) were filled with RPMI 1640 medium supplemented with 1% fetal calf serum (FCS, Merck, Darmstadt, Germany). The migration of cells was monitored using a time-lapse microscope (Lumascope LS620, Etaluma, Carlsbad, CA, USA), at 37 °C, in a 5% CO_2_ atmosphere, for 24 hours. The series of time-lapse images was analyzed using the ImageJ manual-tracking plug-in and Chemotaxis and Migration tool software (ibidi). Three independent experiments were performed.

### 4.4. Transwell Migration Experiments

The migration capacity of ALCL and cHL cell lines towards an FCS gradient was evaluated using transwell inserts (Corning, New York, NY, USA), with an 8 µm pore size. Briefly, 10^5^ cells were plated on the filter in RPMI 1640 medium, containing 1% FCS and 1% penicillin/streptomycin, and cells were allowed to migrate to a lower chamber filled with RPMI 1640 medium, containing 20% FCS and 1% penicillin/streptomycin, for 20–22 hours. After the indicated period of time, the cells in the lower chamber were counted and assessed for viability with trypan blue staining. Three independent experiments were performed.

### 4.5. Co-Culture Experiments and Cluster Formation Assay

On day 0, CD4^+^ T cells purified from peripheral blood mononuclear cells of healthy donors (see Supplementary Methods) were labeled with 2 µM CellTracker red dye (Molecular Probes, Thermo Fisher, Waltham, MA, USA). Lymphoma cell lines were labeled with 1 µM CellTrace carboxyfluorescein succinimidyl ester (CFSE) dye (Thermo Fisher) in serum-free RPMI 1640 with Glutamax medium (Gibco, Thermo Fisher), at 37 °C, in the dark. CD4^+^ T cells were mixed with tumor cells at a 10:1 ratio and then cultured overnight. Five µg of anti-human CD2 antibody (BD Pharmingen, San Jose, CA, USA) or 10 µg anti-CD58 TS2/9 antibody (Thermo Fisher) were added to some wells. On day 1, the cluster formation, as assessed by fluorescent color overlay, was analyzed with a Lumascope LS620 (Etaluma) at 40× magnification. Then, FCS in the medium was adjusted to a concentration of 10%. On day 4, the effects of the blocking antibodies on cluster formation were analyzed using brightfield microscopy (Lumascope LS620, Etaluma) at 10× magnification, and three images per well were taken. The clusters were counted and measured using ImageJ software. Three independent experiments were performed.

### 4.6. Analysis of Filopodia-Like Structures in a Collagen Type I Gel

Life-Act-GFP expressing ALCL cell lines (DEL and SUDHL-1) and cHL cell lines (L-428 and L-1236) were plated in a 1.5 mg/mL bovine collagen type I gel on an Ibidi Microslide Angiogenesis (Ibidi). The cells were equilibrated for 1 h in a cell culture incubator. Z-stack 3D images were acquired from Life-Act -GFP expressing cell lines using a spinning disc microscope with a 63× oil objective at a 0.3 µm interval. Segmentation of the cells was performed using the successive combination of a global threshold for the complete picture and local thresholds for each cell. Cells touching each other were separated using the watershed algorithm. The filopodia-like structures, which were Life-Act-GFP positive structures emerging from the cell membrane, were segmented using a difference of Gaussian (DoG) filter to enhance the signal, followed by global thresholding and skeletonization to isolate the actin filaments (for details see Supplementary Methods). A total of 36–56 GFP-Life-Act-labeled cells (all cells per image) were analyzed each per cell line in four independent experiments by spinning disk microscopy.

### 4.7. Statistical Analysis

Data were tested for the presence of a Gaussian distribution (Shapiro–Wilk-test). If present, groups were compared using the unpaired or paired t-test; otherwise, the Mann–Whitney test was applied. For multiple comparisons, a One-Way Anova with Bonferroni´s multiple comparison test or Kruskal-Wallis-test was performed. Variance can differ between groups, however none of the used test needs equal variances as a precondition. Plots in Figure 2 show medians, minimums and maximums. Figure 4A shows means and standard deviation. All other plots show means and standard error of means.

## 5. Conclusions

In the present study, we found several factors, which explain the differences in clinical presentation between ALCL and cHL. We here confirm the secretion of the chemokines CCL17 and CCL22 by HRS cells of cHL, resulting in the attraction of T cells, which are closely attached to HRS cells by adhesion molecules. The larger sizes of HRS cells, combined with the surrounding T-cell rosette, can restrain the dissemination of these cells in the preformed lymphoid sinuses of a lymph node. Additionally, we could for the first time demonstrate differences in intrinsic basal cell motility between cHL and ALCL cell lines contributing to a less efficient spread of HRS cells than ALCL cells. 

## Supplementary Materials

The following are available online at https://www.mdpi.com/2072-6694/11/10/1484/s1, File S1: materials and methods. Figure S1: Max Length of filopodia-like structures, Figure S2: Collagen cleavage in ALCL and cHL cell lines, Figure S3: Application of global thresholds onto the preprocessed picture, Table S1: Number of evaluable cases with tumor cells positive for selected chemokine receptors (immunohistochemistry), Table S2: Flow cytometric analysis of chemokine receptor expression in ALCL and cHL cell lines, Table S3: Immunohistochemical analysis of expression of chemokines by tumour cells in ALCL and cHL cases, Table S4: Expression of chemokines in ALCL and cHL cell lines determined with a human chemokine array. Video S1: DEL cells (ALCL) transduced to express Life-Act (green). Nuclei are stained with SIR-DNA (red). 100× objective, Video S2: L-1236 cells (cHL) transduced to express Life-Act (green). Nuclei are stained with SIR-DNA (red). 100× objective.
