# Supplementary material for: Migration Properties Distinguish Tumor Cells of Classical Hodgkin Lymphoma from Anaplastic Large Cell Lymphoma Cells
Source: Cancers (Basel). 2019 Oct 2;11(10):1484. doi: 10.3390/cancers11101484 (PMC6827161; doi:10.3390/cancers11101484)
Supplement: Supplementary file 1 [file cancers-11-01484-s001.zip › Description of Supplementary Movies S1 and S2.docx]

**Supplementary Movie S1. DEL cells (ALCL) transduced to express Life-Act (green). Nuclei are stained with SIR-DNA (red). 100x objective.**

**Supplementary Movie S2. L-1236 cells (cHL) transduced to express Life-Act (green). Nuclei are stained with SIR-DNA (red). 100x objective.**
