# Supplementary material for: Migration Properties Distinguish Tumor Cells of Classical Hodgkin Lymphoma from Anaplastic Large Cell Lymphoma Cells
Source: Cancers (Basel). 2019 Oct 2;11(10):1484. doi: 10.3390/cancers11101484 (PMC6827161; doi:10.3390/cancers11101484)
Supplement: Supplementary file 1 [file cancers-11-01484-s001.zip › Supplemental materials and methods.docx]

**Supplemental materials and methods**

Immunohistochemistry

Antigen unmasking was performed for 2 min in a pressure cooker in TRIS EDTA at pH 8.0. Tissue sections were incubated with primary antibodies (anti-CD4: 1:50, DAKO, Glostrup, Denmark, anti-CD8: 1:100, DAKO, anti-CD163: 1:100 DAKO) for 30 minutes at room temperature. For detection the K5005 kit (DAKO) was used. Immunohistochemical stainings were quantified after scanning and digitalization of images with an Aperio Scanner as described previously.^1^ For each slide, three representative counting frames (122,824 mm2) were chosen, and the number of positively stained cells was counted. For immunohistochemical stainings of CCRs, the following antibodies were applied: CCR5: GTX109635, Genetex, CCR4: A4427, Lifespan, both antigen unmasking at pH6, antibody dilution 1:100, incubation for 2 hrs at room temperature. CCR1: A1242, Lifespan antigen unmasking at pH8, antibody dilution 1:200, incubation for 2 hrs at room temperature CXCR3: TA340421, Origene, antigen unmasking at pH8, antibody dilution 1:50, incubation for 2 hrs at room temperature. All antibodies recognizing CCRs were detected by the Envision FLEX Detection System (DAKO).

Cell culture

ALCL cell lines SU-DHL-1, SUP-M2 (DSMZ, Braunschweig, Germany) and cHL cell lines L-1236, L-540 (DSMZ) were cultured in RPMI 1640 cell culture medium (Sigma, Merck, Darmstadt, Germany) with Glutamax (Gibco, Thermofisher, Darmstadt, Germany) supplemented with 20% FCS and 1% penicillin/streptomycin (Gibco). ALCL cell lines DEL, MAC-1, KARPAS 299, TS-G1 and cHL- cell lines L-428, and KM-H2 were cultured in RPMI 1640 cell culture medium with Glutamax supplemented with 10% FCS and 1% penicillin/streptomycin. ALCL cell line SR-786 was cultured in RPMI 1640 cell culture medium with Glutamax supplemented with 15% FCS and 1% penicillin/streptomycin. Cell culture was splitted every 2-3 days with an exchange of medium.

Flow cytometry analysis

The cells were stained with APC-conjugated antibodies recognizing chemokine receptors: CCR1, CXCR3, CCR4 or CCR5 (Miltenyi Biotec, Bergisch Gladbach, Germany) with Fc blocking reagent human (Miltenyi Biotec) and analyzed with the flow cytometer MACSQuant (Miltenyi Biotec).

Lentiviral vectors and transduction of ALCL and cHL cell lines

The lentiviral vector pLenti.PGK.LifeAct-GFP.W was a gift from Rusty Lansford (Addgene plasmid # 51010; http://n2t.net/addgene:51010 ; RRID:Addgene_51010). Lentiviral vector supernatants were produced as described previously.^2^ ALCL and cHL cell suspensions (cell concentration 2x10^6^/ml) were incubated with lentiviral supernatants for 48 hours.

Analysis of cell motility in a collagen type I gel

The cell line of interest (prepared in suspension at a density of 18x10^6^ cells/ml in RPMI 1640 medium supplemented with 10% fetal calf serum (FCS) (Merck). After gelation of a 1.5 mg/ml bovine collagen type I gel (Advanced Biomatrix, San Diego, CA, USA), left and right chambers of Ibidi µ-slide chemotaxis (Ibidi, Martinsried, Germany) were filled with RPMI 1640 supplemented with 1% FCS (Merck). The migration of cells was monitored using the Lumascope LS620 (Etaluma, Carlsbad, CA, USA) at 37^o^C in an atmosphere with 5% CO_2_ for 24 hours. The series of time-lapse images was analyzed using ImageJ manual-tracking plug-in and Chemotaxis and Migration tool software (ibidi). In total, approximately 40 trajectories per cell line were analyzed per experiment. Only cells with a velocity ≥ 0.1 µm/h were considered as moving cells and cells with a velocity ≤ 0.1 µm/h were excluded from further analysis.

Isolation of CD4-T cells from peripheral blood

Peripheral blood mononuclear cells (PBMCs) were obtained by gradient Ficoll-Paque Plus (GE Healthcare, Uppsala, Sweden) centrifugation of peripheral blood of healthy donors. CD4 T cells were separated with MACS using the human CD4 T cells isolation kit (Miltenyi Biotec). The purity of the CD4 T cell population was confirmed via FACS analysis using an anti-human CD4-APC antibody (Miltenyi Biotec). Cells with a purity >90% were used for further experiments.

Coculture experiments and cluster formation assay

At day 0, CD4^+^ T cells purified from PBMCs were labeled with 2 µM CellTracker red dye (Molecular Probes, Thermo Fisher). ALCL cell lines (SU-DHL-1, DEL), cHL cell lines (L-1236, L-428) and the Burkitt lymphoma cell line Ramos were labeled with 1 µM CellTrace CFSE dye (Thermo Fisher) in serum-free RPMI 1640 with Glutamax medium (Gibco, Thermofisher) at 37^o^C in the dark. CD4^+^ T cells were mixed with ALCL, cHL or Burkitt lymphoma cells at a ratio 10:1 and then cultured in RPMI 1640 medium with Glutamax supplemented with 1% FCS and 1% penicillin/streptomycin (Gibco, Thermofisher) overnight. Five µg of anti-human CD2 antibody (BD Pharmingen, San Jose, CA, USA) or 10 µg anti-CD58 TS2/9 antibody (Thermo Fisher) were added to some wells. At day 1, the cluster formation as fluorescent color overlay was analyzed with a Lumascope LS620 (Etaluma, Carlsbad, CA, USA) at 40x magnification. Then, the amount of FCS in the medium was adjusted to 10%. At day 4, the effect of blocking antibodies on cluster formation was analyzed using brightfield microscopy (Lumascope LS620, Etaluma) at 10x magnification and three images per well were taken. The clusters were counted and measured using ImageJ software.

Quantification of pericellular collagenolysis

ALCL cell lines (DEL and SU-DHL-1: 8x10^3^ cells each), cHL cell lines (L-428 and L-1236: 8x10^3^ cells each) as well as fibrosarcoma cell line HT-1080 (3.2x10^3^ cells) were embedded in a 1.5 mg/ml bovine collagen type I gel in Ibidi microslide angiogenesis (ibidi) according to manufacturer's instructions. The slides were kept in the incubator overnight in order to allow the cells to migrate. Next day, the gel was washed with DPBS (Gibco, Thermofisher) and then fixed using 4% paraformaldehyde (PFA) for 30 minutes at 37°C. Staining of the cleaved collagen was performed with anti-Col1-3/4C antibody (collagen type I cleavage site) (Immunoglobe, Himmelstadt, Germany) primary antibody and goat anti-rabbit Alexa Fluor 647 (Invitrogen, Thermo Fisher) secondary antibody. The nuclei of the cells were counterstained with DAPI (Sigma, Merck). The z-stacks of a cleaved collagen were acquired with 10x objective and 4 µm interval using a Spinning disk microscope (Zeiss, Oberkochen, Germany). In order to quantify the amount of collagen digestion, the picture was divided into fore- and background. The foreground (FG) contained the position of the cells and their neighborhood and was detected by applying the Otsu threshold (doi:10.1109/TSMC.1979.4310076) onto the DAPI channel. Onto the resulting binary picture the Dilate operation (radius: 5 pixel) was carried out to not only include cell nuclei but also the surrounding area. The background (BG) contained the remaining parts of the picture. Then the average fluorescence in the collagen channel was computed for both areas and finally the difference between both areas is computed (fluorescence BG - fluorescence FG).

Segmentation of cells in a collagen type I gel

Segmentation of the cells was performed using a successive combination of a global threshold for the complete picture and local thresholds for each cell, because (1) single cells have different fluorescence intensities due to varying expression levels of Life-Act-GFP and (2) the background around cells with high expression show higher intensity values compared to the body of cells with low expression. Cells touching each other were separated using the watershed algorithm. For all the analyses, cells which were located on the border of the image were excluded. Segmentation of cells is written completely in python using the skimage package^3^ for image processing. During preprocessing the image is first scaled to get a uniform pixel size in x, y and z dimension. Then the 3D image is filtered with a gauss filter (sigma: 1.6) to remove noise and is converted to a 2D image using a maximum intensity projection. Afterwards a gamma transformation (gamma: 0.3) is applied to equalise the intensity values of all cells and background/uneven exposure is removed of the image by subtracting a gaussian filtered image with a very large radius (sigma:150). Now we still observed the problem that the background intensity around bright cells can be higher as the intensity of some other cells. As a result the triangle thresholds^4^ wrongly classifies some background pixels as foreground and more strict thresholds like for example the Li threshold^5^ fails to classify all cells as foreground (Supplementary Figure S3). To evade this problem we implemented a two step thresholding procedure: (1) To detect all cells a global triangle threshold is applied to the complete picture and small holes are closed using the open operation for binary pictures (radius: 6). Then a marker based watershed algorithm is applied to the distance transform of the binary picture to split touching cells into different objects. As markers the local maxima of the distance transform image, with a height corresponding at least to the minimal fragment radius (6 pixels), are used. (2) To detect the true border of all cells we applied a lokal-like threshold for each cell. In order to do so, we first enlarged each cell area with an open operation (radius: 20) to ensure that background is present in the pixel set. Now a Li threshold is applied for the single cell and its direct surrounding. This procedure is carried out for all cells individually based on their average intensity value in a decreasing order. After having the final classification of background and foreground pixels a second round of a marker based watershed algorithm on the distance transformed binary image is performed to again split touching cells. This time markers are forced to be separated by at least 40 pixels, which orients on the minimal diameter of cells. To obtain only the cell body and to exclude bunches of filopodia which are detected by this procedure, we use the open operation for each single cell with the largest possible disk shaped structure element which does not remove the complete cell.

After segmentation of all pictures the Jaccard index (Jindex = TP/(TP+FP+FN); TP = true positive, FP = false positive, FN, false negative) was determined automatically for all pictures of one replica and all values are above 0.9 (Del: 0.97, SU-DHL-1: 0.98, L-428: 0.94, L-1236: 0.96), indicating a good segmentation. The assignment of automatically and manually detected cell centres, used to compute the Jaccard index, is obtained with the help of the linear assignment problem applied to a full distance matrix (between manually and automatically segmented cell centers), constructed in analogy to TrackMate.^6^

Segmentation of Life-Act positive actin structures

Also the segmentation of actin structures is written completely in python using the skimage package^3^ for image processing. The filopodia-like structures, which are Life-Act-GFP positive structures emerging from the cell membrane, were segmented using a difference of Gaussian (DoG) filter to first enhance the signal, followed by a global thresholding and a skeletonization to obtain the actin filaments. This time the uniformly scaled 3D picture is filtered with a gauss filter (sigma: 1.0) to remove noise and is also converted to a 2D image using a maximum intensity projection. Similar to the Filoquant software^7^ we segmented the actin filaments using skeletonization: To enhance the signal of thin actin structures a difference of gaussian filter (sigma1: 0.5, sigma2: 1.5) was applied and all negative values were set to zero. Subsequently the actin structures are binarised using a mean threshold and the binary image is skeletonized in order to extract the overall structure of the actin filaments. Actin filaments with a length under 4 pixels were excluded from the analysis. To measure the length of the skeleton branches the skan package^8^ is used. A filopodium is assigned to a cell if one part is inside the segmented area of the complete cell (including the filopodia bunches). To analyse the orientation of the actin structures in relation to the orientation of the cell membrane, we computed the angle between two vectors. The first one is defined by the start and end position of the filopodium and the second is defined by the cell mid point and the starting point of the filopodium. For a better visualization of the nuclei, nuclei were stained by SIR-DNA in some experiments that were not included in the systematic analysis of actin filaments (Supplementary Movies 1 and 2).

**References**

1. Scheidt V, Hansmann ML, Schuhmacher B, et al. Atypical variants of nodular lymphocyte predominant Hodgkin lymphoma show low microvessel density and vessels of distention type. Human Pathology. 2016.

2. Weiser C, Petkova MV, Rengstl B, et al. Ectopic expression of transcription factor BATF3 induces B-cell lymphomas in a murine B-cell transplantation model. Oncotarget. 2018;15942-15951.

3. Pedregosa F, Varoquaux G, Gramfort A, et al. Scikit-learn: Machine Learning in Python. Journal of Machine Learning Research. 2011.

4. Zack GW, Rogers WE, Latt SA. Automatic measurement of sister chromatid exchange frequency. J Histochem Cytochem. 1977;741-753.

5. Li CH, Lee CK. Minimum cross entropy thresholding. Pattern Recognition. 1993;617-625.

6. Tinevez JY, Perry N, Schindelin J, et al. TrackMate: An open and extensible platform for single-particle tracking. Methods. 2017;80-90.

7. Jacquemet G, Paatero I, Carisey AF, et al. FiloQuant reveals increased filopodia density during breast cancer progression. J Cell Biol. 2017;3387-3403.

8. Nunez-Iglesias J, Blanch AJ, Looker O, et al. A new Python library to analyse skeleton images confirms malaria parasite remodelling of the red blood cell membrane skeleton. PeerJ. 2018;e4312.
