# Supplementary material for: Migration Properties Distinguish Tumor Cells of Classical Hodgkin Lymphoma from Anaplastic Large Cell Lymphoma Cells
Source: Cancers (Basel). 2019 Oct 2;11(10):1484. doi: 10.3390/cancers11101484 (PMC6827161; doi:10.3390/cancers11101484)
Supplement: Supplementary file 1 [file cancers-11-01484-s001.zip › Supplementary Table S1.docx]

**Supplementary Table S1. Number of evaluable cases with tumor cells positive for selected chemokine receptors (immunohistochemistry)**

| **Receptor** | **ALK^–^ ALCL (%)** | **ALK^+^ ALCL (%)** | **cHL (mixed cellularity) (%)** |
| --- | --- | --- | --- |
| **CXCR3** | 8/13 (62%) | 6/11 (55%) | 5/11 (45%) |
| **CCR4** | 9/15 (60%) | 10/15 (67%) | 6/10 (60%) |
| **CCR5** | 17/22 (77%) | 12/16 (75%) | 6/8 (75%) |
| **CCR1** | 1/23 (4%) | 3/13 (23%) | 4/10 (40%) |
