# Supplementary material for: Migration Properties Distinguish Tumor Cells of Classical Hodgkin Lymphoma from Anaplastic Large Cell Lymphoma Cells
Source: Cancers (Basel). 2019 Oct 2;11(10):1484. doi: 10.3390/cancers11101484 (PMC6827161; doi:10.3390/cancers11101484)
Supplement: Supplementary file 1 [file cancers-11-01484-s001.zip › Supplementary Table S2 R1.docx]

**Supplementary Table S2. Flow cytometric analysis of chemokine receptor expression in ALCL and cHL cell lines.**

| **Cell lines** | **CXCR3** | **CCR4** | **CCR5** | **CCR1** |
| --- | --- | --- | --- | --- |
| ALCL cell lines |  |  |  |  |
| **SUDHL-1 (ALK^+^)** | - | - | + | - |
| **DEL (ALK^+^)** | - | - | - | - |
| **SR786 (ALK^+^)** | + | - | - | - |
| **KARPAS-299 (ALK^+^)** | - | - | - | - |
| **MAC-1 (ALK^-^)** | - | + | + | - |
| cHL cell lines |  |  |  |  |
| **L-428 (nodular sclerosis)** | - | + | - | - |
| **L-1236 (mixed cellularity)** | - | + | - | - |
| **L-540 (nodular sclerosis)** | - | - | - | - |
| **KM-H2 (mixed cellularity)** | - | + | - | - |

A cell line was considered as positive when at least 30% of cells expressed the respective chemokine receptor.
