# Supplementary material for: Migration Properties Distinguish Tumor Cells of Classical Hodgkin Lymphoma from Anaplastic Large Cell Lymphoma Cells
Source: Cancers (Basel). 2019 Oct 2;11(10):1484. doi: 10.3390/cancers11101484 (PMC6827161; doi:10.3390/cancers11101484)
Supplement: Supplementary file 1 [file cancers-11-01484-s001.zip › Supplementary Table S3 R1.docx]

**Supplementary Table S3. Immunohistochemical analysis of expression of chemokines by tumour cells in ALCL and cHL cases**

| **Chemokine analysed** | **Corresponding CCR** | **ALK^-^ ALCL** **(%)** | **ALK^+^ ALCL** **(%)** | **cHL (mixed cellularity subtype) (%)** |
| --- | --- | --- | --- | --- |
| **CXCL9** | CXCR3 | 7/9 (78%) | 5/7 (71%) | 4/ 10* (40%) |
| **CXCL10** | CXCR3 | 5/9 (55%) | 2/7 (29%) | 4/16 (25%) |
| **CCL17** | CCR4 | 0/23 (0%) | 0/12 (0%) | 17/20 (85%) |
| **CCL22** | CCR4 | 0/20 (0%) | 0/10 (0%) | 48/60** (80%) |
| **CCL3** | CCR5, CCR1 | 5/16 (31%) | 5/17 (29%) | n.d. |
| **CCL4** | CCR5 | 5/9 (55%) | 1/8 (13%) | n.d. |
| **CCL5** | CCR5, CCR1 | 3/18 (17%) | 6/19 (32%) | 12/14 (86%) |

n.d. not done

* Data previously published in Hartmann et al., Int J Cancer 2013

** Data previously published in Döring et al., Mod Pathol 2014. This study includes both cHL of the mixed cellularity and nodular sclerosing type. No differences in CCL22 expression were observed between both cHL subtypes.
