# Supplementary material for: Migration Properties Distinguish Tumor Cells of Classical Hodgkin Lymphoma from Anaplastic Large Cell Lymphoma Cells
Source: Cancers (Basel). 2019 Oct 2;11(10):1484. doi: 10.3390/cancers11101484 (PMC6827161; doi:10.3390/cancers11101484)
Supplement: Supplementary file 1 [file cancers-11-01484-s001.zip › Supplementary Table S4.docx]

| **Chemokine** | **ALCL cell lines** | | | | | | | **cHL cell lines** | | | |
| --- | --- | --- | --- | --- | --- | --- | --- | --- | --- | --- | --- |
|  | SUDHL-1 | DEL | MAC-1 | SR-786 | KARPAS-299 | TS-G1 | SUP-M2 | L-428 | L-1236 | L-540 | KM-H2 |
| **CCL21** | - | - | - | - | - | - | - | - | - | - | - |
| **CCL28** | - | - | - | - | - | - | - | - | - | - | - |
| **CXCL16** | - | - | **+** | - | - | - | - | - | - | **+** | - |
| **TIG-2** | - | - | - | - | - | - | - | - | - | - | - |
| **CXCL5** | - | - | - | - | - | - | - | - | - | - | - |
| **CCL26** | - | - | - | - | - | - | - | - | - | - | - |
| **CX3CL1** | - | - | **-** | - | - | - | - | **+** | - | - | **+** |
| **CXCL1** | - | - | - | - | - | - | - | - | - | - | - |
| **CCL14** | - | - | - | - | - | - | - | - | - | - | - |
| **CCL1** | - | - | **+** | - | - | - | - | - | - | - | - |
| **CXCL8** | - | **+** | - | - | - | **+** | **+** | - | - | - | - |
| **LCF** | - | - | - | - | - | - | - | - | - | - | - |
| **CXCL10** | **+** | - | **+** | - | - | - | - | - | - | - | **+** |
| **CXCL11** | - | - | - | - | - | - | - | - | - | - | - |
| **XCL1** | - | - | - | - | - | - | - | - | - | - | - |
| **CCL2** | - | - | - | **-** | **-** | **-** | **-** | - | **+** | **-** | **-** |
| **CCL7** | - | - | - | - | - | - | - | - | - | - | - |
| **CCL22** | - | - | - | **-** | **-** | **-** | **-** | - | **+** | **-** | **-** |
| **CXCL9** | - | - | - | - | - | - | - | - | - | - | - |
| **CCL3,CCL4** | - | - | **+** | **-** | **-** | **-** | **-** | - | **+** | **-** | **-** |
| **CCL15** | - | - | - | - | - | - | - | - | - | - | - |
| **CCL20** | - | **+** | - | - | - | - | - | - | - | - | - |
| **CCL19** | - | - | - | - | - | - | - | - | - | - | - |
| **CXCL7** | - | - | - | - | - | - | **+** | - | - | - | - |
| **CCL18** | - | - | - | - | - | - | - | - | - | - | - |
| **CXCL4** | - | - | - | - | - | - | - | - | - | - | - |
| **CCL5** | - | - | **+** | **-** | **-** | - | **-** | **+** | **+** | **+** | **+** |
| **CXCL12** | **+** | - | **+** | - | - | - | - | - | - | - | - |
| **CCL17** | - | - | **-** | **-** | **-** | **-** | **-** | **+** | **+** | **-** | **+** |
| **CXCL17** | - | - | - | - | - | - | - | - | - | - | - |
| **Midkine** | - | - | - | - | - | - | - | - | - | **+** | - |

**Supplementary Table S4.** Expression of chemokines in ALCL and cHL cell lines determined with a human chemokine array. One array per cell line was performed.
