# Supplementary figures and images for: Migration Properties Distinguish Tumor Cells of Classical Hodgkin Lymphoma from Anaplastic Large Cell Lymphoma Cells
Source: Cancers (Basel). 2019 Oct 2;11(10):1484. doi: 10.3390/cancers11101484 (PMC6827161; doi:10.3390/cancers11101484)

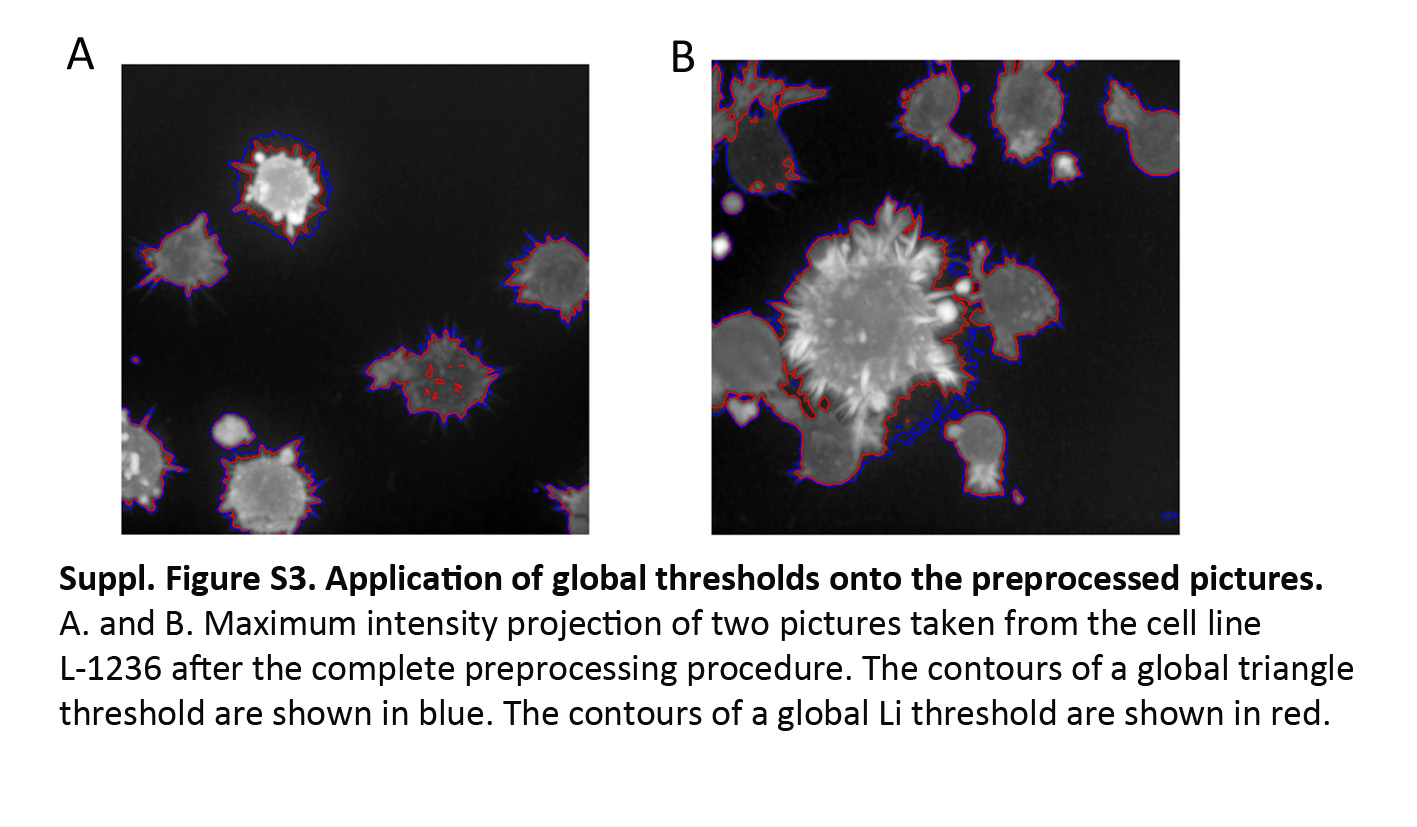

Supplement: Supplementary file 1 [file cancers-11-01484-s001.zip › Suppl. Figure S3.jpg]

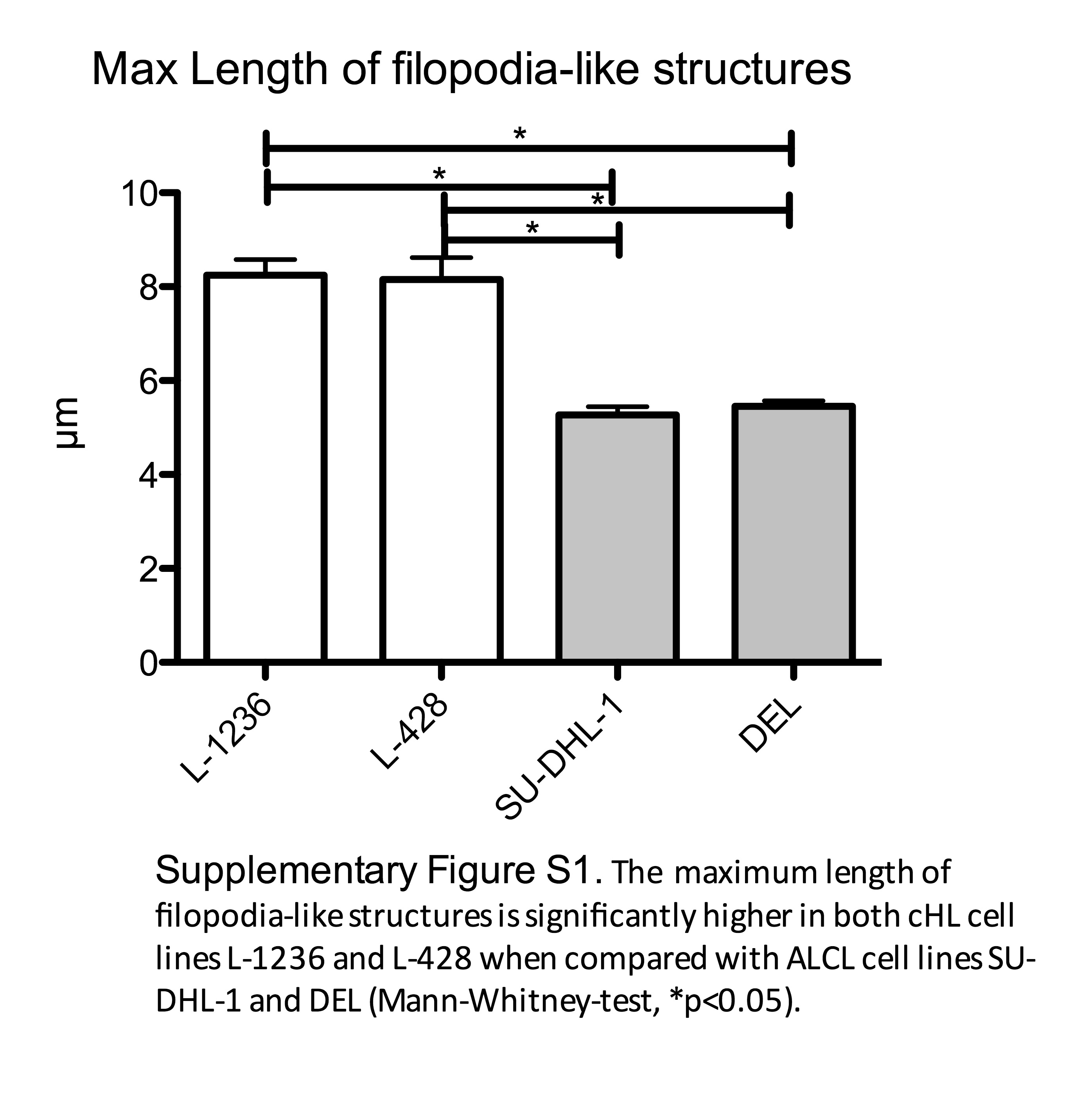

Supplement: Supplementary file 1 [file cancers-11-01484-s001.zip › Supplementary Figure S1.jpg]

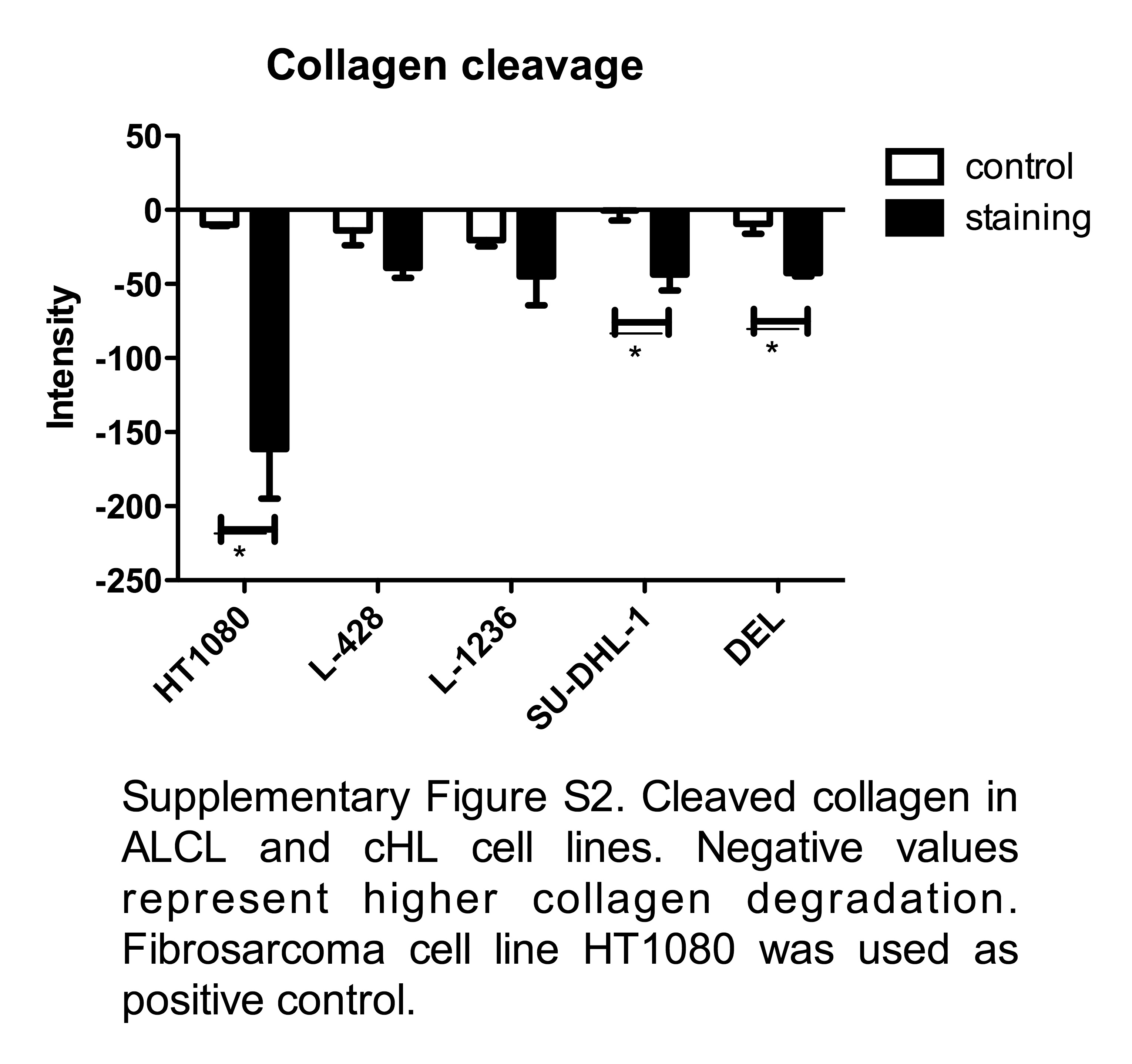

Supplement: Supplementary file 1 [file cancers-11-01484-s001.zip › Supplementary Figure S2.jpg]
